# CMOS-compatible synaptic transistor gated by chitosan electrolyte-Ta_2_O_5_ hybrid electric double layer

**DOI:** 10.1038/s41598-020-72684-2

**Published:** 2020-09-23

**Authors:** Shin-Yi Min, Won-Ju Cho

**Affiliations:** Department of Electronic Materials Engineering, Kwangwoon University, Chambit-Kwan, B 104, Wolgye 1-dong, Nowon-gu, Seoul, 139-701 Korea

**Keywords:** Electrical and electronic engineering, Bioinspired materials

## Abstract

This study proposes a hybrid electric double layer (EDL) with complementary metal-oxide semiconductor (CMOS) process compatibility by stacking a chitosan electrolyte and a Ta_2_O_5_ high-*k* dielectric thin film. Bio-inspired synaptic transistors with excellent electrical stability were fabricated using the proposed hybrid EDL for the gate dielectric layer. The Ta_2_O_5_ high-*k* dielectric layer with high chemical resistance, thermal stability, and mechanical strength enables CMOS-compatible patterning processes on biocompatible organic polymer chitosan electrolytes. This technique achieved ion-conduction from the chitosan electrolyte to the In-Ga-Zn oxide (IGZO) channel layer. The on/off current ratio, subthreshold voltage swing, and the field-effect mobility of the fabricated IGZO EDL transistors (EDLTs) exhibited excellent electrical properties of 1.80 × 10^7^, 96 mV/dec, and 3.73 cm^2^/V·s, respectively. A resistor-loaded inverter was constructed by connecting an IGZO EDLT with a load resistor (400 MΩ) in series. This demonstrated good inverter action and responded to the square-wave input signals. Synaptic behaviours such as the hysteresis window and excitatory post-synaptic current (EPSC) variations were evaluated for different DC gate voltage sweep ranges and different AC gate spike stimuli, respectively. Therefore, the proposed organic–inorganic hybrid EDL is expected to be useful for implementing an extremely compact neural architecture system.

## Introduction

Neural systems have been actively investigated to replace traditional Boolean logic and von Neumann architecture^1,2^. Inspired by biological neurons and synapses, neural systems have many distinctive advantages, including massive parallelism, power-efficiency, storage/computation combination, and self-learning^3,4^. Two-terminal memristors, which use conducting filaments, phase change materials, and protonic layers between two electrodes, have been proposed for artificial synaptic devices through the various neural functions and geometric advantages^5–7^. However, cross-point array memristors are suppressed by the sneak current issue, in which the current flows through the unselected cells, and this can lead to low energy efficiency and excessive noise problems^8,9^. In contrast, multi-terminal synaptic devices are more appropriate for complex neural networks and real-time processing due to their additional gate terminals. When the voltage spike stimulus is applied to the gate terminals, the channel conductance between the source and the drain terminals is modulated. Therefore, three-terminal synaptic transistors can be simultaneously realised for the functions of learning/memory with a high energy efficiency^1,10,11^.

In the past few years, three-terminal synaptic transistors that are gated by ion-conducting electrolytes have attracted significant attention. The ion-conducting electrolytes are reasonable for the electric-double-layer (EDL) modulation, and they enable the EDL transistors (EDLTs) to achieve synaptic dynamic functions. The most important property of the EDL is to act like a nanogap capacitor (typical EDL thickness is ~ 1.0 nm). The specific capacitance of the EDL can be extremely large (~ μF/cm^2^) due to the EDL effect and the induced high-carrier density is up to 10^15^ cm^−2^. Therefore, the modulated EDL can precisely trigger an ionic excitatory post-synaptic current (EPSC) to control the synaptic plasticity for low power consumption, which is a connection strength between two neighbouring synapses^12–15^.

Meanwhile, recent interest in using natural biomaterials has received significant attraction because they are eco-friendly and biocompatible for electronic systems. Chitosan is a natural cationic biopolymer that is derived from chitin, which is composed of repeated *β*(1,4)-linked d-glucosamine (*N*-deacetylated chitin) and *N*-acetyl-d-glucosamine units^16,17^. Chitosan-based bio-electrolyte is promising based on the following advantages, and it is widely investigated for the EDL. (1) Chitosan is the second most abundant polysaccharide on Earth and is extracted from the shells of crabs and shrimp; (2) Chitosan is an inexpensive, non-toxic, biocompatible polymer; (3) Chitosan is a low-cost solution that can achieve processability; (4) Chitosan has high-transparency and flexibility for its medium molecular weight; (5) Finally, chitosan has high-capacitance for protonic mobile ions^10,18,19^. In previous studies, numerous studies of chitosan electrolyte-based synaptic transistors, such as chitosan electrolyte-based metal–oxide–semiconductor channel-synaptic transistors on transparent glass substrate^19,20^, synaptic transistors on flexible freestanding chitosan-based membranes^10,21^, chitosan electrolyte-based SnO_2_ nanowire channel synaptic transistors^22^, and two-dimensional MoS_2_ channel-synaptic transistors^23^, have been extensively reported. This highly applicable chitosan electrolyte could offer not only artificial synaptic device applications but also versatile engineering platforms like skin-attachable, wearable, bio-sensor, and digestible smart electronics. However, despite the advantages of chitosan-based EDL, organic electrolyte-based EDLTs are hard to apply for the conventional complementary metal-oxide semiconductor (CMOS) process. This is attributed to their low chemical resistance, thermal, and ambient instabilities^24^.

Therefore, in this study, we propose an organic–inorganic hybrid EDL. This is achieved by stacking a Ta_2_O_5_ high-*k* thin film on a solution-processed chitosan electrolyte layer to fabricate three-terminal synaptic transistors with excellent electrical characteristics and CMOS process compatibility. The excellent chemical resistance, thermal stability, and mechanical strength of the inorganic Ta_2_O_5_ film allowed the CMOS-compatible patterning process of the organic polymer chitosan electrolyte layer to obtain small feature size EDLTs. The transfer and output characteristics of the fabri19cated organic–inorganic hybrid IGZO EDLT were evaluated. In addition, by configuring an IGZO EDLT-based resistor-loaded inverter with load resistors that are connected in series, we verified the static voltage transfer characteristics and dynamic inverting characteristics. Finally, we evaluated the synaptic behaviours, such as the change in the hysteresis voltage for the direct current (DC) gate voltage sweep range and the variation in the EPSC for the alternating current (AC) gate spike stimulus.

## Results

### Photolithography process on chitosan electrolyte-Ta_2_O_5_ hybrid EDL

**Figure 1:**
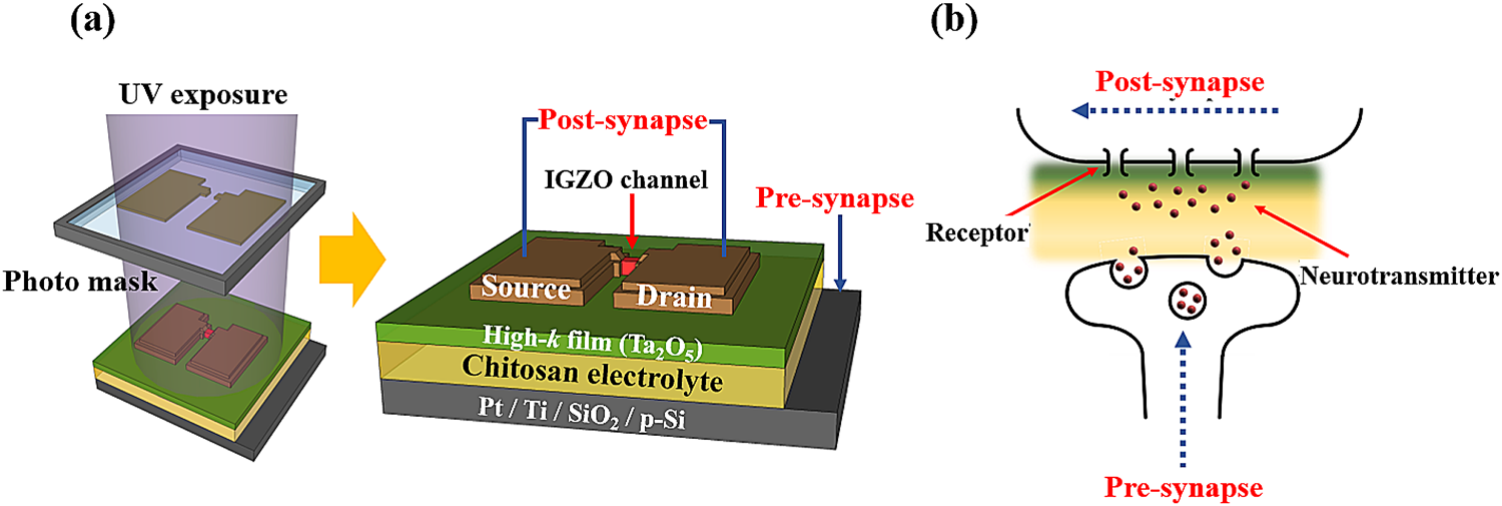
Schematic of (**a**) the bottom-gate top-contact structure chitosan electrolyte-Ta_2_O_5_ hybrid EDLTs fabricated by the photolithography process and (**b**) the simplified biological synapse.

Figure 1a shows a schematic of the fabricated bottom-gate top-contact structure chitosan electrolyte-Ta_2_O_5_ hybrid EDLT. The process of patterning the IGZO active channel and the top-contact source/drain (S/D) Ti electrodes was followed using a photolithography process for the chitosan electrolyte-Ta_2_O_5_ hybrid EDL. The dimensions of the fabricated EDLT are 20 μm for the channel width (W_CH_) and 10 μm for the channel length (L_CH_). Figure 1b illustrates a simplified schematic of a biological synapse. When the synaptic signal (stimulus) reaches the pre-synaptic neuron (Pt bottom-gate), it releases neurotransmitters from the pre-synapse. Subsequently, the receptors capture the neurotransmitters, and this can lead to the potential change in the post-synaptic neuron (IGZO channel)^1, 25^.

In the organic–inorganic hybrid EDLT that is displayed in Fig. 1a, the organic polymer chitosan electrolyte functions as a neurotransmitter to mimic the synaptic behaviour. However, despite the remarkable imitation properties for the synapses, it is hard to apply a photolithography process that is essential for device integration on an organic electrolyte layer due to swelling and an insufficient chemical resistance^18,21,24,26^. Although the chitosan patterning process in a previous study was conducted by electrodeposition or mould casting that was used at both micro and macro levels^27^. we developed a CMOS-compatible patterning process by performing photolithography.

**Figure 2:**
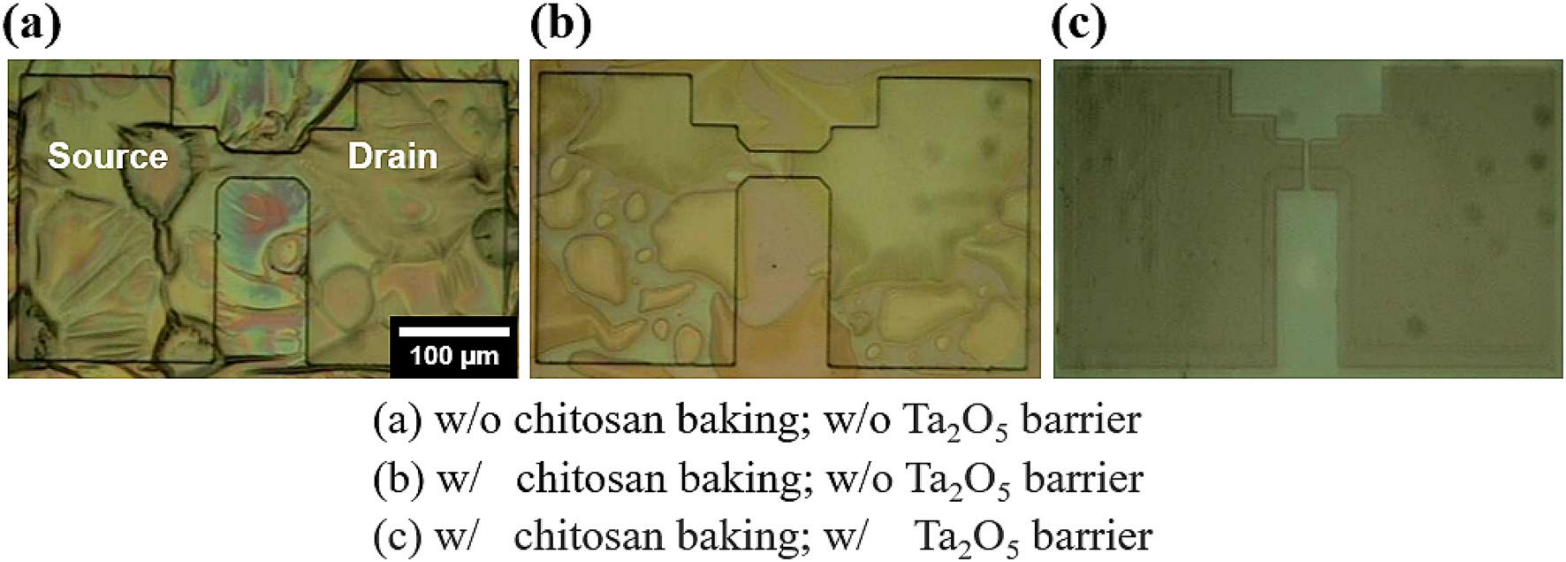
Optical microscopic images of the EDLTs fabricated by the lithography process. (**a**) Without chitosan baking and without the Ta_2_O_5_ thin film. (**b**) With chitosan baking and without the Ta_2_O_5_ thin film. (**c**) With chitosan baking and with the Ta_2_O_5_ thin film. (**a**) and (**b**) show the deformed EDL, and (**c**) shows the normal EDL.

Figure 2 shows the optical microscope images of the fabricated bottom-gate top-contact EDLTs. In the absence of the Ta_2_O_5_ high-k thin film, as shown in Fig. 2a,b, the EDL undergoes swelling or outgassing during baking or photolithography. This indicates that the chemical resistance of the chitosan electrolyte is insufficient. In contrast, as shown in Fig. 2c, the hybrid EDL with the Ta_2_O_5_ high-k thin film on the baked organic polymer chitosan layer was well prepared without swelling or outgassing during photolithography (see Supplementary Information Figure S1). Accordingly, it has been determined that the Ta_2_O_5_ high-k thin film acts as an effective barrier to prevent deformation of the organic polymer chitosan layer to ensure a stable CMOS patterning process.

### Electrical properties of the chitosan electrolyte-Ta_2_O_5_ hybrid EDLT

**Figure 3:**
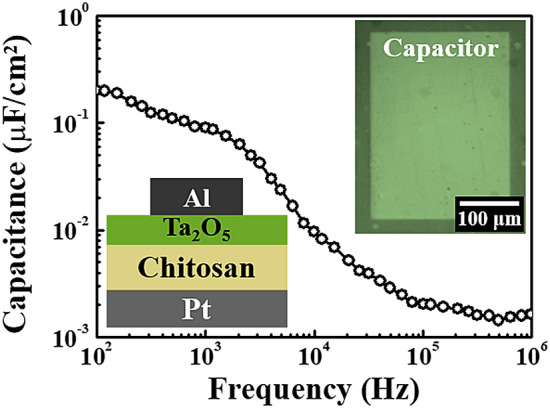
Capacitance–frequency (C–*f*) curve for the chitosan electrolyte-Ta_2_O_5_ hybrid EDLC that is measured in the frequency range from 10^2^ to 10^6^ Hz. The insets show the schematic and the optical microscope image of the hybrid EDLC.

To evaluate the dynamic behaviour of the chitosan electrolyte, the capacitance–frequency (C–*f*) curve characteristics of the Al/Ta_2_O_5_/chitosan/Pt structure EDL capacitor (EDLC) were measured in the frequency range of 10^2^–10^6^ Hz. As a result, a specific capacitance profile is clearly expressed for the specified frequency range as shown in Fig. 3. As demonstrated, the capacitance increases with a decreasing frequency, and the maximum capacitance of ~ 0.2 μF/cm^2^ was obtained at a frequency of 10^2^ Hz. This strong frequency-dependent capacitance can be originated from the EDL effect through the mobile ions within the chitosan electrolyte^23,28–30^. There are many mobile ions in the chitosan electrolyte, which includes anions and cations.

**Figure 4:**
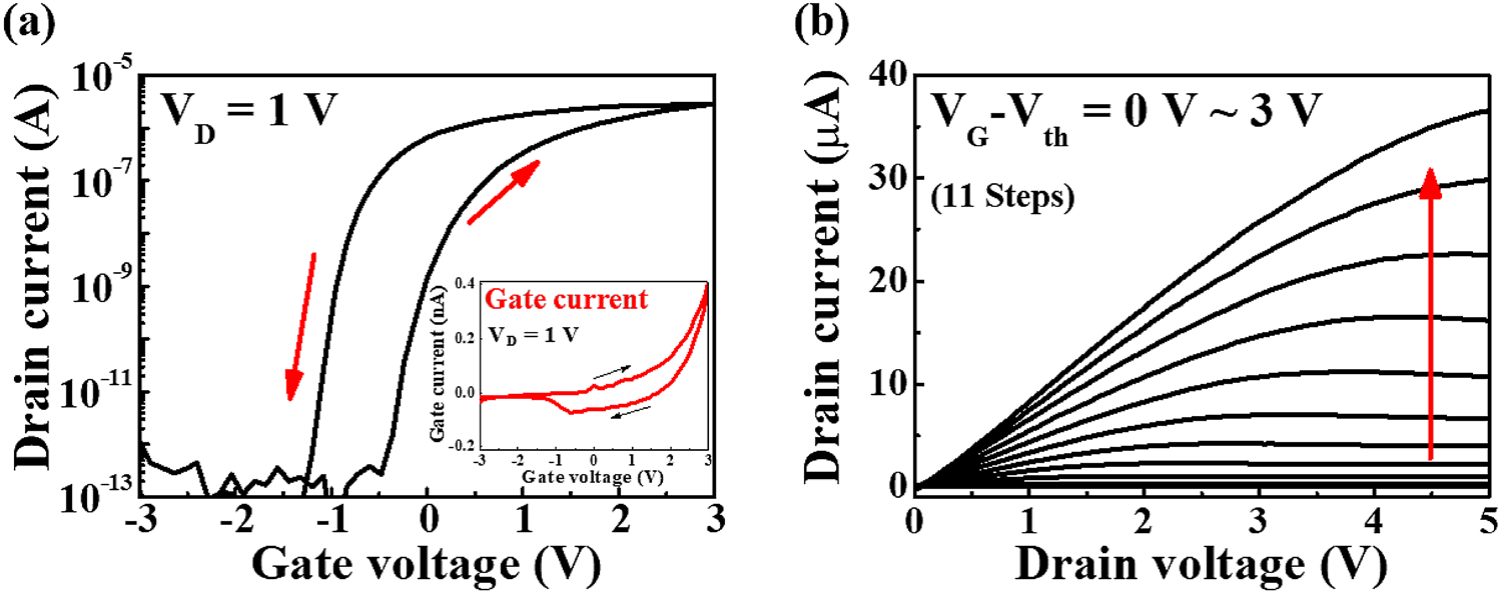
Electrical properties of the chitosan electrolyte-Ta_2_O_5_ hybrid EDLT. (**a**) The transfer characteristics, where V_G_ was swept from – 3 to 3 V in the dual-sweep mode at V_D_ = 1 V. The inset shows the gate leakage current. (**b**) The output characteristics (I_D_–V_D_) measured by V_G_–V_th_ from 0 to 3 V in 0.3 V steps.

Figure 4a shows the transfer characteristics (I_D_–V_G_) of the chitosan electrolyte-Ta_2_O_5_ hybrid EDLT. Moreover, to evaluate the hysteresis characteristics of the EDLT, the drain current (I_D_) was measured for the dual-sweep mode with a drain voltage (V_D_) of 1 V, the gate voltage (V_G_) was swept from – 3 to 3 V, and back to – 3 V. The threshold voltage (V_th_) was obtained by extrapolating the linear curve for the transfer characteristics, which was – 0.19 V; this exhibited a high on/off current ratio of about 1.8 × 10^7^. The subthreshold voltage swing (SS) and field-effect mobility (μ_FE_) were 96 mV/dec and 3.73 cm^2^/V·s, respectively, and these were obtained by the following equations:

1 $$SS={\left[{\left(\frac{dlog{I}_{D}}{d{V}_{G}}\right)}_{max}\right]}^{-1}$$

and

2 $${\mu }_{FE}=\frac{L}{W\cdot {C}_{i}\cdot {V}_{D}}\cdot \frac{d{I}_{D}}{d{V}_{G}}$$

where L is the channel length, W is the channel width, and C_i_ is the gate capacitance per unit area from the EDLC. In addition, the hysteresis window (ΔV_th_) was defined as the difference between the threshold voltages for the forward sweep (V_th_^f^) and the backward sweep (V_th_^b^), ΔV_th_ = V_th_^f^–V_th_^b^. As a result, a slow polarisation reaction by the mobile ions in the chitosan electrolyte induces a counter-clockwise hysteresis and the ΔV_th_ is 0.92 V^18^. The inset of Fig. 4a is the gate current of the chitosan electrolyte-Ta_2_O_5_ hybrid type EDL in the transfer operation, which shows a low leakage current of 0.4 nA at 3 V. Figure 4b shows the output characteristics (I_D_–V_D_) that are measured by V_G_–V_th_ from 0 to 3 V by performing steps that are 0.3 V. In the low V_D_ region, the I_D_ linearly increases with an increasing voltage, which indicates ohmic contact between the IGZO channel layer and the metal (Ti) S/D electrode. In addition, as the V_D_ further increases, the I_D_ gradually becomes saturated and it exhibits pinch-off characteristics.

**Figure 5:**
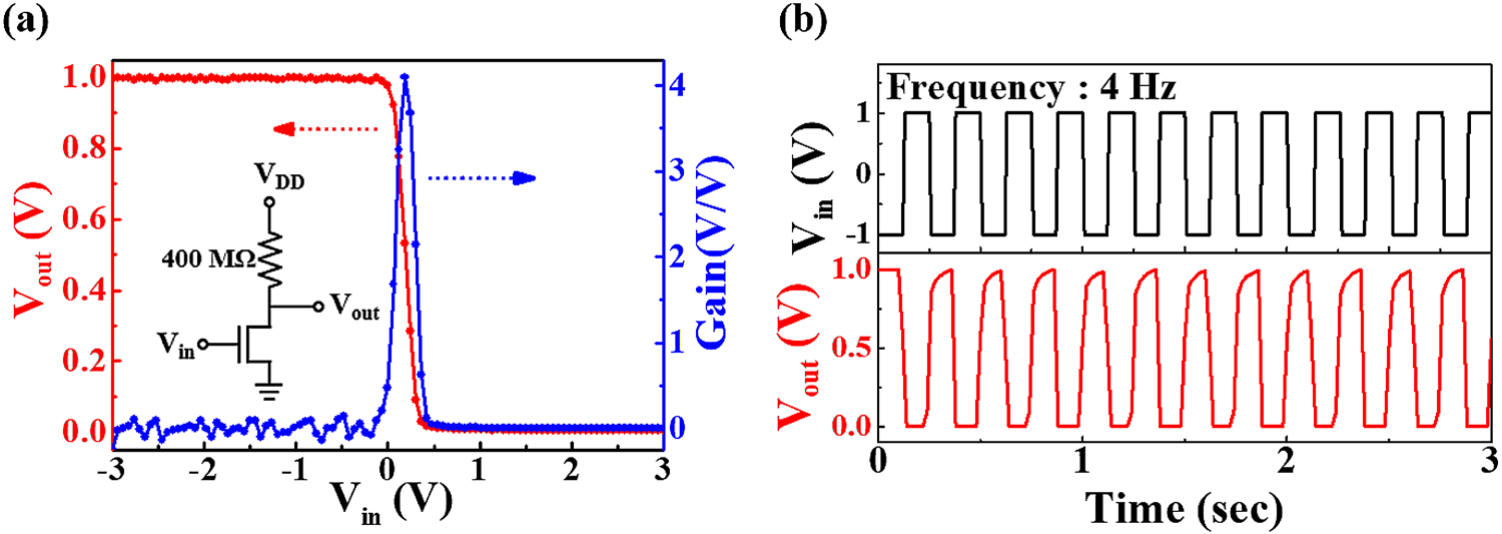
Resistor-loaded inverter characteristics of the chitosan-Ta_2_O_5_ hybrid EDLT. (**a**) The VTC curve and voltage gain were obtained by connecting a load resistor of 400 MΩ in series (V_DD_ = 1 V). The inset is a simple schematic of an equivalent circuit for a resistor-loaded inverter. (**b**) The dynamic inverting response properties at a frequency of 4 Hz.

Figure 5 shows the static voltage transfer characteristics (VTC) and the dynamic inverting characteristics measured from a simple resistor-loaded inverter circuit. This was built by connecting a load resistor of 400 MΩ in series to a chitosan electrolyte-Ta_2_O_5_ hybrid type EDLT. As shown in the equivalent circuit of the inset in Fig. 5a, the source electrode of the EDLT was fixed at a ground voltage, and the supply voltage (V_DD_) of 1 V was applied to a resistor that was connected in series to the drain electrode. Figure 5a shows the VTC curve of the inverter with the input voltage (V_in_) applied to the pre-synapse gate electrode. When the V_in_ is low (V_in_ <  − 0.1 V), the driver EDLT is in the “OFF” state, which was obtained with a high output (V_out_) of ~ 1 V. However, when V_in_ is relatively high (V_in_ > 0.4 V), the driver EDLT is in the “ON” state, which was obtained with a low V_out_ of ~ 0 V. It can be observed that an abrupt voltage transition of V_out_ occurs in the VTC curve in response to V_in_ at ~ 0.3 V. The voltage gain (–dV_out_/dV_in_) from the VTC curve of the resister-loaded inverter is ~ 4.1 (V_DD_ = 1 V). Figure 5b is a dynamic inverting response of the resistor-loaded inverter, which switches between − 1 V and + 1 V in response to a square-wave V_in_ with a frequency of 4 Hz (V_DD_ = 1 V). The resistor-loaded inverter exhibits good inverting action and well responds by following a low-power square-wave input signal. Such relaxation time along the order of milliseconds appeared in the output signal is known to be related to the migration and the accumulation of protons in the chitosan electrolyte^19,30^. This long relaxation time is a drawback in traditional logic circuit applications, but it is rather favourable in the operation of low-power artificial synaptic electronics, which suggests potential applications^13,31,32^.

### Synaptic operations of the chitosan electrolyte-Ta_2_O_5_ hybrid EDLT

**Figure 6:**
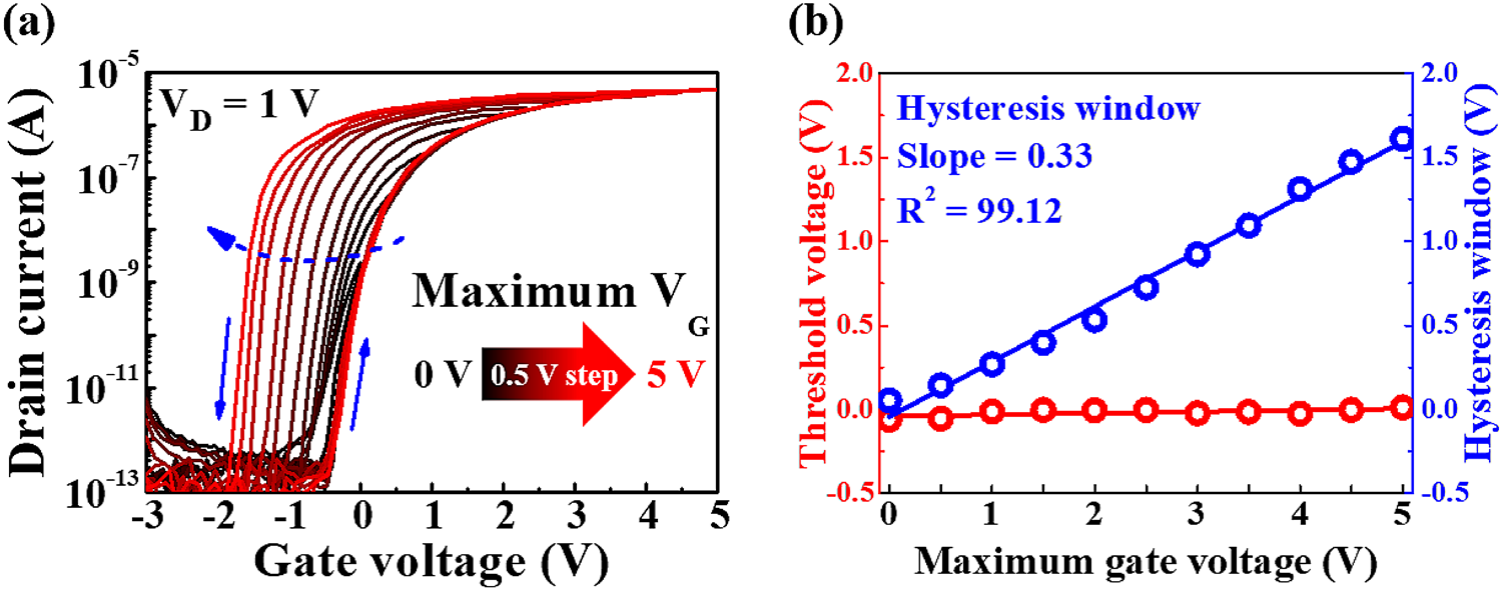
(**a**) Transfer characteristic curves of the chitosan electrolyte-Ta_2_O_5_ hybrid EDLT according to the V_G_ sweep range. Maximum V_G_ = 0 to 5 V, in 0.5 V increments. (**b**) Dependence of the hysteresis window and the V_th_ on the maximum V_G_.

The transfer characteristics presented in Fig. 4a display a counter-clockwise hysteresis window that is induced by the mobile ions in the chitosan electrolyte. To identify the magnitude of the polarisation response due to the migration of the mobile ions, we evaluated the transfer characteristics of the chitosan electrolyte-Ta_2_O_5_ hybrid type EDLT according to the V_G_ sweep range. Figure 6a shows the change in the counter-clockwise hysteresis window of the transfer characteristics that is measured in the dual-sweep mode according to the V_G_ sweep range (V_D_ = 1 V). It can be observed that the hysteresis window increases as the maximum V_G_ increases from 0 to 5 V in 0.5 V increments. Figure 6b shows the V_th_ and hysteresis window that is extracted from the transfer characteristic curves for the maximum V_G_. As the maximum V_G_ increased from 0 to 5 V, V_th_ remained almost constant, while the hysteresis window exhibited a linear increase from 0.05 to 1.61 V. The relationship between the maximum V_G_ and the hysteresis window has high linearity (> 99%) with a slope of 0.33 V/V. The polarisation of the dipoles in the Ta_2_O_5_ high-*k* dielectric and the migration of mobile ions in the chitosan electrolyte induce charge carriers at the interface of the IGZO channel/hybrid type EDL. In addition, changes in the gate electric field can cause dipole alignment and migration of the mobile ions. The larger the maximum V_G_, the larger the electric field, which can result in a stronger dipole alignment and ion movement. This makes it difficult to quickly return to the original state during the backward V_G_ sweep. This leads to a decrease in V_th_ and an increase in I_D_; thus, resulting in greater hysteresis for the transfer characteristics during the dual-sweep mode. This hysteretic phenomenon is initialised by applying a relatively large negative V_G_ =  − 3 V, where the dipoles and migrated mobile ions are fully depolarised, and they release the charge carriers from the conductive channel. Therefore, the drain current of the chitosan-Ta_2_O_5_ hybrid EDLT returns to the initial off state. These initialisation properties and linear relationships of the maximum V_G_ vs hysteresis window indicate that the proposed device can mimic the biological synapse^33–35^.

**Figure 7:**
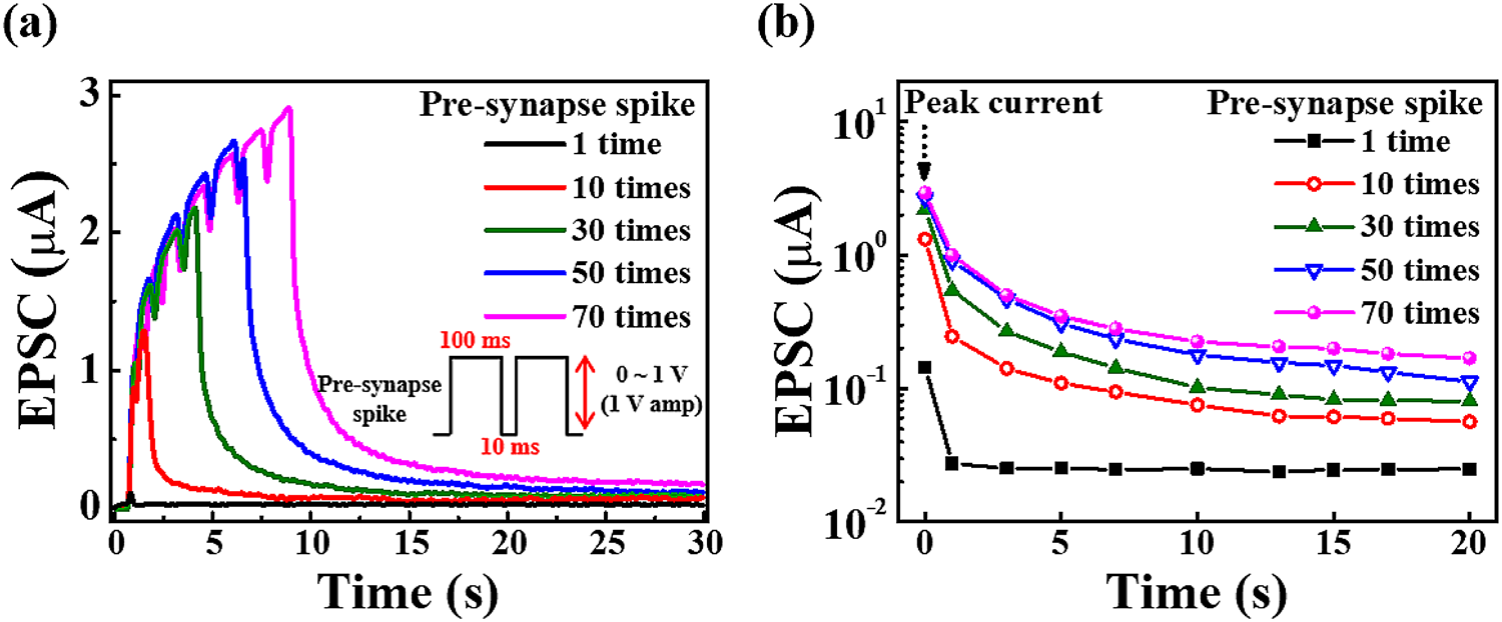
(**a**) EPSC response according to the number of pre-synaptic stimulus spikes (1 V, 100 ms). (**b**) The decay of the EPSC stimulated by a different number of pre-synaptic gate pulses.

In the biological neural systems, synaptic plasticity is considered as the function for the mimicking synaptic operations of the EDLTs. In Fig. 1b, the mobile ions in the chitosan electrolyte are used as the neurotransmitters, which deliver the spike stimulus from the pre-synapse to the post-synapse. Therefore, the EPSC of the IGZO channel can be modulated by the synaptic weight^36,37^. To evaluate this behaviour for the biological synaptic operation of the chitosan electrolyte-Ta_2_O_5_ hybrid type EDLTs, a pre-synaptic spike (1 V, 100 ms) was applied to the bottom-gate electrode and a readout voltage (V_D_ = 1 V) for the EPSC measurement was applied between the S/D electrodes. Figure 7a shows the EPSC response according to the number of pre-synaptic stimulus spikes. When a single spike pulse is applied to the gate, the EPSC reaches a peak level ≈ 140 nA at the end of the stimulus, and then it rapidly decays to a resting current level ≈ 25 nA in milliseconds. This short EPSC duration time represents the short-term potentiation (STP). This occurs as a concentration gradient due to the migration of mobile ions by the stimulus, as it returns to equilibrium in a short time. In contrast, when multiple spike pulses are applied to the gate, the magnitude of the EPSC steadily increases with the number of spikes until the final stimulus, and then it decays gradually. Figure 7b shows the decay of the EPSC after the spike stimulus over time with respect to the number of spike pulses. It can be determined that the EPSC successively increases as the number of gate pulses increases. In contrast to the response after a single spike pulse, the EPSC after multiple spike pulses did not decay to the resting current level after 20 s from the peak current. This long EPSC duration time represents the long-term potentiation (LTP). Furthermore, as the number of pulses increases, the peak current and the resting current levels increase. By having a greater number of pulses, there is a greater concentration gradient of mobile ions in the chitosan electrolyte (ion charging process) and this increases the time to return to equilibrium (ion discharge process)^10,18,36^. In addition, there is a large number of traps due to the structural imperfections at the chitosan/Ta_2_O_5_ interface. As the number of protons increases with an increase in the number of pulses, the number of protons captured at this interface also increases; thus, resulting in a longer EPSC duration.

**Figure 8:**
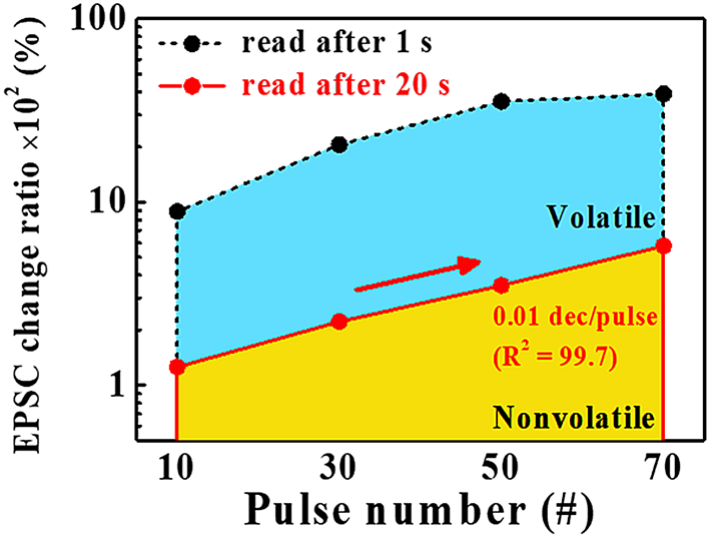
Dependence of the EPSC change ratio, ((*I*–*I*_0_)/*I*_0_ × 100%), according to the pulse spike numbers. *I*_0_ is the resting current and *I* is the EPSC in the channel after the pre-synaptic stimulus.

The residual EPSC after 20 s over time is significantly enhanced with an increase in the spike pulse numbers, which indicates a different trend from the STP to the LTP. Figure 8 shows the pulse number dependence of the EPSC change ratio, ((*I* – *I*_0_)/*I*_0_ × 100%), where *I*_*0*_ is the resting current and *I* is the EPSC of the channel after the pre-synaptic stimulus. The rate of the EPSC change increases with the number of pulses in the volatile and non-volatile regions. In addition, the non-volatile region is gradually increased with the number of pulses, which follows the linear relationship respect to the EPSC change ratio slope of 0.01 dec/pulse number (R^2^ = 99.7). This suggests the possibility of biocontrol for the magnitude of the STP and the LTP, which can be used as short-term and long-term memory, respectively, at artificial synapses^1,36,38^.

## Conclusion

This study proposes bottom-gate top-contact structure synaptic transistors that are gated by an organic–inorganic hybrid EDL. The CMOS-compatible patterning processes on the organic polymer chitosan electrolytes were successfully conducted through the stacking of a biodegradable chitosan electrolyte and the Ta_2_O_5_ high-*k* dielectric thin film. The value of the chitosan electrolyte-Ta_2_O_5_ hybrid EDL capacitance is increased by decreasing the frequency. In addition, a large capacitance ~ 0.2 μF/cm^2^ was obtained at 10^2^ Hz. The on/off current ratio, V_th_, SS, and μ_FE_ values of the chitosan electrolyte-Ta_2_O_5_ hybrid EDLT were 1.80 × 10^7^, – 0.19 V, 96 mV/dec, and 3.73 cm^2^/V·s, respectively. In addition, they exhibit an abrupt voltage transition in the VTC and good dynamic inverting actions to the low-power square-wave input signals for the resistor-loaded inverter circuit. In terms of the biological synaptic operation, the chitosan electrolyte-Ta_2_O_5_ hybrid EDLT represents a linear polarisation magnitude relationship through the maximum V_G_ with a constant V_th_. Finally, the EPSC is modulated by the pre-synaptic stimulus numbers and it has an increased portion of the STP and LTP because the stimulus number is increasing. As a result, the EDLT gated by the chitosan electrolyte-Ta_2_O_5_ hybrid EDL is expected to provide compact neural architecture systems. This can be achieved by applying a CMOS-compatible photolithography process with excellent electrical properties and synaptic operations.

## Methods

### Solution synthesis procedure

The solution of the chitosan electrolyte was synthesised by the sol–gel reaction of the chitosan powder and acetic acid. The dried form of the chitosan powder and the flakes are typically insoluble in distilled water. The shrimp shell-based chitosan powder with a medium molecular weight (deacetylation degree > 75%, Sigma Aldrich) was dissolved (2 wt%) in 2% acetic acid solution (> 99%, Sigma Aldrich), which was diluted with distilled water. Subsequently, the solution was mixed under 800 rpm at 50 ℃ in a constant magnetic stirring system for 6 h. Subsequently, the solution was filtered through a polytetrafluoroethylene (PTFE) syringe filter with 5-μm pore-size to remove the particulates.

### Device fabrication procedure

The bottom-gate top-source/drain structure chitosan electrolyte-Ta_2_O_5_ hybrid EDLTs were fabricated as follows. The 300 nm-thick thermally grown oxide on the (100)-orientated p-type silicon wafer was used as a starting material, and it was cleaned by a standard RCA process. First, the 10-nm-thick Ti and 100 nm-thick Pt were sequentially deposited by using an E-beam evaporator for a bottom-gate (pre-synapse). Second, to form the chitosan electrolyte-Ta_2_O_5_ hybrid EDL, the chitosan solution was spin-coated and dried at room temperature (25 °C) for 24 h. Then, the samples were oven-baked at 130 °C for 10 min to remove the residual moisture in the chitosan electrolyte film. In sequence, an 80 nm-thick Ta_2_O_5_ high-*k* dielectric was deposited on a 130-nm-thick chitosan layer by the radio frequency (RF) magnetron sputtering system with a 20 sccm-Ar flow rate, 3.0 mTorr-working pressure, and a 75 W-RF power. Third, the 50 nm-thick IGZO channel layer was also deposited by the RF magnetron sputtering system with a 30 sccm-Ar flow rate, 6.0 mTorr-working pressure, and a 100 W-RF power. The active patterning process of the IGZO channel layer was performed by a positive type photolithography technique and a wet etching process with a 30:1 ratio for the buffer oxide etchant. Finally, 100-nm-thick Ti source/drain electrodes (post-synapse) were deposited by the E-beam evaporator and they were patterned through the lift-off method. The defined channel width and length were 20 μm and 10 μm, respectively. The fabricated chitosan electrolyte-Ta_2_O_5_ hybrid EDLTs were positioned at room temperature (25 °C) in a dark box to avoid light and electrical noise. The electrical characteristics and synapse operations of the fabricated devices were investigated using the Agilent 4156B Precision Semiconductor Parameter Analyser and they were pulsed by the Agilent 8110A Pulse Generator. In addition, the C-*f* curve characteristic of the Al/Ta_2_O_5_/chitosan/Pt structure EDLC was measured at the frequency range of 10^2^ to 10^6^ Hz by using Agilent 4284A Precision LCR Meter.

## Supplementary information

Supplementary file
