# Supplementary material for: CMOS-compatible synaptic transistor gated by chitosan electrolyte-Ta2O5 hybrid electric double layer
Source: Sci Rep. 2020 Sep 23;10:15561. doi: 10.1038/s41598-020-72684-2 (PMC7511302; doi:10.1038/s41598-020-72684-2)
Supplement: Supplementary file 1 — Supplementary file [file 41598_2020_72684_MOESM1_ESM.docx]

CMOS-compatible synaptic transistor gated by chitosan electrolyte-Ta_2_O_5_ hybrid electric double layer

Shin-Yi and Won-Ju Cho^*^

Department of Electronic Materials Engineering, Kwangwoon Univ. Chambit-kwan, B 104, Wolgye 1-dong, Nowon-gu, Seoul 139-701, Korea

^*^E-mail: [chowj@kw.ac.kr](mailto:chowj@kw.ac.kr)

**Supplementary Information**

**1. Oven-baking temperature of the chitosan electrolyte**

**Figure S1** shows the optical microscope (× 300) images of the chitosan electrolyte-Ta_2_O_5_ hybrid-type films. **Figures S1a–d** depict the as-prepared chitosan electrolyte-Ta_2_O_5_ hybrid-type films at the different chitosan electrolyte oven-baking temperatures of 50 °C, 100 °C, 130 °C, and 180 °C, respectively. Hot-plate baking is essential for the photoresist in the photolithography process and **Figures S1e–h** show the corresponding images to **Figures S1a–d** after hot-plate baking at 120 °C for 2 min. In **Figures S1f–h**, there were no significant changes after hot-plate baking for the chitosan electrolyte-Ta_2_O_5_ hybrid films. However, in **Figure S1e**, the 50 °C oven-baked chitosan electrolyte shows an outgassing phenomenon due to the residual moisture of the chitosan electrolyte.


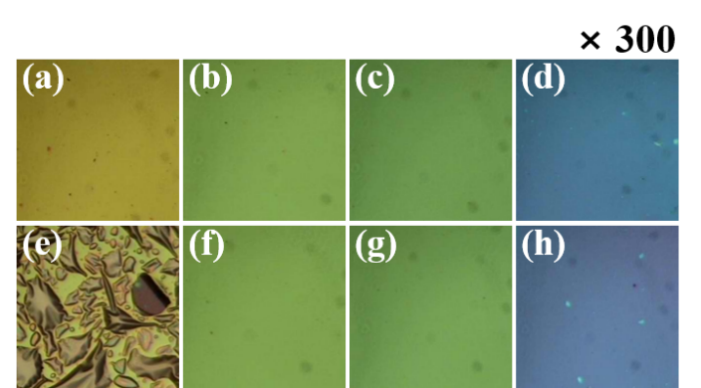


**Figure S1.** Optical microscope (× 300) images of the as-prepared chitosan electrolyte-Ta_2_O_5_ hybrid film at the different chitosan electrolyte oven-baking temperatures: (a) 50°C, (b) 100°C, (c) 130°C, and (d) 180°C. The images in (e) to (h) correspond to the samples in (a) to (d) after hot-plate baking at 120 °C for 2 min, respectively.

**2. Electrical properties of the chitosan electrolyte-Ta_2_O_5_ hybrid EDLTs**

**Figure S2** shows the transfer characteristics (I_D_–V_G_) of the chitosan electrolyte-Ta_2_O_5_ hybrid EDLTs according to the different chitosan electrolyte oven-baking temperatures of 100 °C, 130 °C, and 180 °C, respectively. The gate voltage was applied on the bottom electrode and the drain voltage was constant at 1 V. The chitosan electrolyte-Ta_2_O_5_ hybrid EDLT with the 130 °C-baked chitosan electrolyte shows the best electrical performance. In contrast, the 100 °C-baked device shows a negatively shifted threshold voltage (V_th_) and the 180 °C-baked device has a smaller on/off current ratio compared with the 130 °C-baked chitosan electrolyte-Ta_2_O_5_ hybrid EDLT.


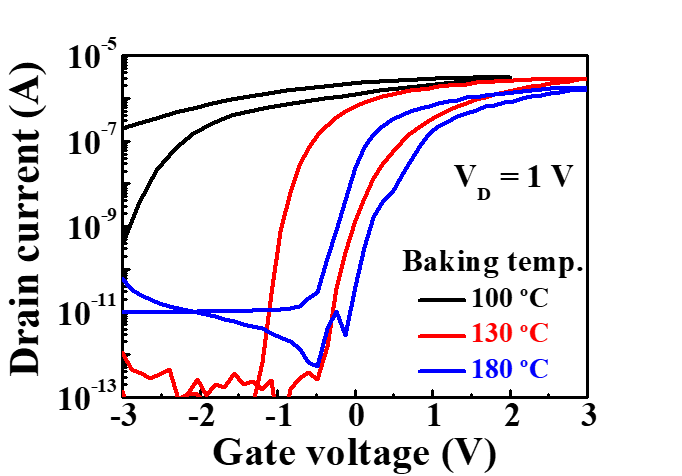


**Figure S2.** The transfer characteristics (I_D_–V_G_) of the chitosan electrolyte-Ta_2_O_5_ hybrid EDLTs according to the different chitosan electrolyte oven-baking temperatures (100 °C, 130 °C, and 180 °C).

**Figure S3** shows the transfer characteristics (I_D_–V_G_) of the chitosan electrolyte-Ta_2_O_5_ hybrid EDLT and only Ta_2_O_5_ layer gated thin-film-transistor (TFT) at a constant drain voltage of 1 V. The only Ta_2_O_5_ (80 nm-thickness) layer gated TFT shows small hysteresis windows (ΔV_th_ = 0.06 V) due to rapid dipoles polarization/depolarization in the Ta_2_O_5_ dielectric layer according to the gate voltage. On the other hand, chitosan electrolyte-Ta_2_O_5_ hybrid EDLT exhibits larger counter-clockwise hysteresis windows (ΔV_th_ = 0.92 V) by the mobile ions in the chitosan electrolyte and lower off-state current than single Ta_2_O_5_ gated TFT.

**
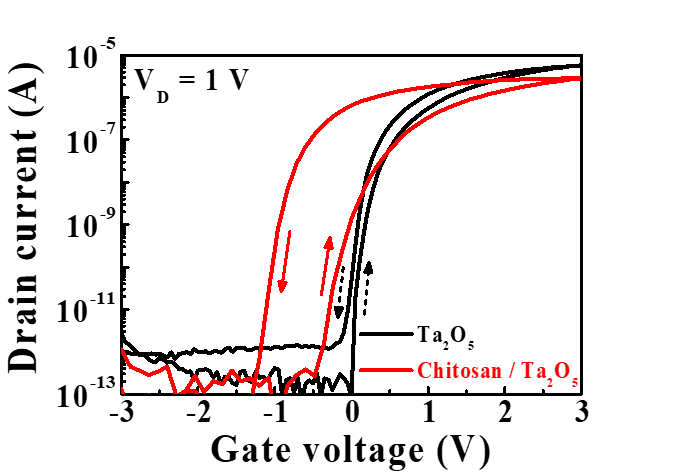
**

**Figure S3.** The transfer characteristics (I_D_–V_G_) of the single Ta_2_O_5_ gated TFT and chitosan electrolyte-Ta_2_O_5_ hybrid EDLT.

**Figure S4** represents the electrical performances of the chitosan electrolyte-Ta_2_O_5_ hybrid EDLTs according to the different Ta_2_O_5_ high-k dielectric layer thicknesses of 30 nm, 80 nm, and 150 nm, respectively. The oven-baking temperature of the chitosan electrolyte was 130 °C for all thicknesses. The chitosan electrolyte-Ta_2_O_5_ hybrid EDLT with the 80 nm-thick Ta_2_O_5_ layer shows the best transfer properties in **Figure S4a**. However, the Ta_2_O_5_ layer with the 30 and 150 nm-thick devices have lower on/off current ratios and smaller hysteresis windows (ΔV_th_). **Figure S4b** shows the output characteristics (I_D_–V_D_) measured by V_G_-V_th_ from 0 to 3 V in 0.3 V steps. The chitosan electrolyte-Ta_2_O_5_ hybrid EDLT with the 80 nm-thick Ta_2_O_5_ layer exhibits a high-drain current with stable linear, pinch-off, and saturation regions. Meanwhile, the 30 nm-thick device shows the unstable drain current drivability and the 150 nm-thick device shows a lower drain current compared to the electrolyte-Ta_2_O_5_ hybrid EDLT with the 80 nm-thick Ta_2_O_5_ layer.


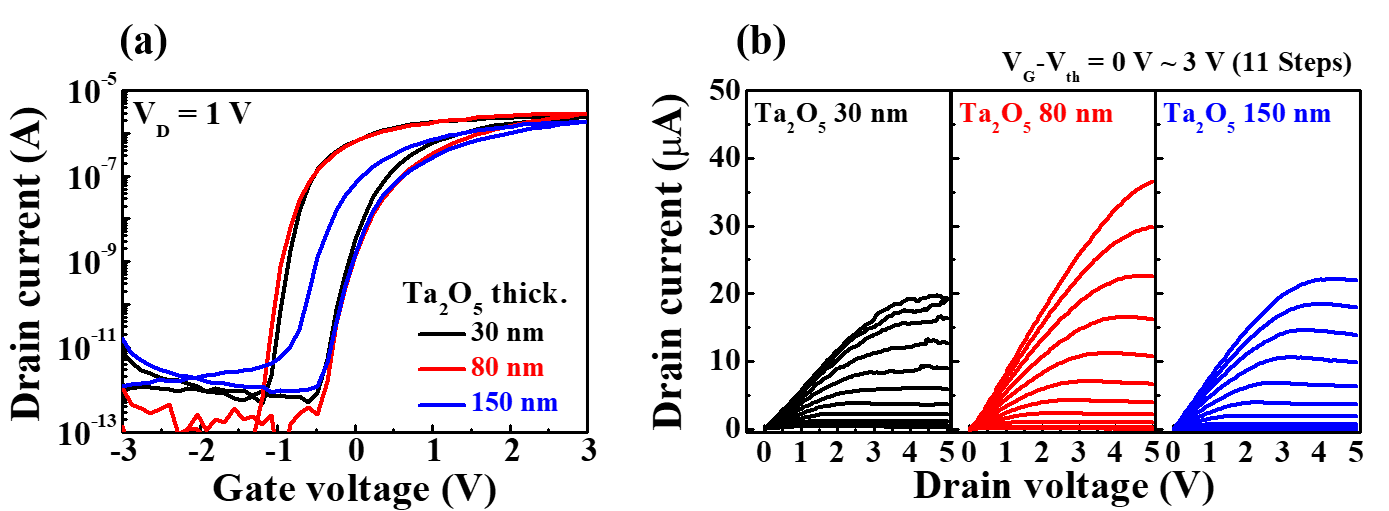


**Figure S4.** Electrical performance of the chitosan electrolyte-Ta_2_O_5_ hybrid EDLTs according to the different Ta_2_O_5_ high-k dielectric layer thicknesses (30 nm, 80 nm, and 150 nm). (a) The transfer characteristics (I_D_–V_G_). (b) The output characteristics (I_D_–V_D_) measured by V_G_–V_th_ from 0 to 3 V in 0.3 V steps.

**3. Inverting characteristics of the chitosan electrolyte-Ta_2_O_5_ hybrid EDLT**

**Figure S5a** represents the static voltage transfer characteristics (VTC) measured from a simple resistor-loaded inverter circuit built by connecting a load resistor (400 MΩ) in series to a chitosan electrolyte-Ta_2_O_5_ hybrid EDLT. The inset in **Figure S5a** displays a simple schematic of the equivalent circuit. The supplied voltage (V_DD_) was applied from 0.2 to 1.0 V (0.2 V steps) and the clear VTC curve was obtained at a 1.0 V magnitude for V_DD_. The voltage gains (–dV_out_/dV_in_) from the VTC curves are represented in **Figure S5b**. The gain values were increased according to the magnitude of V_DD_ and the highest value of ~4.1 was calculated in V_DD_ = 1.0 V.


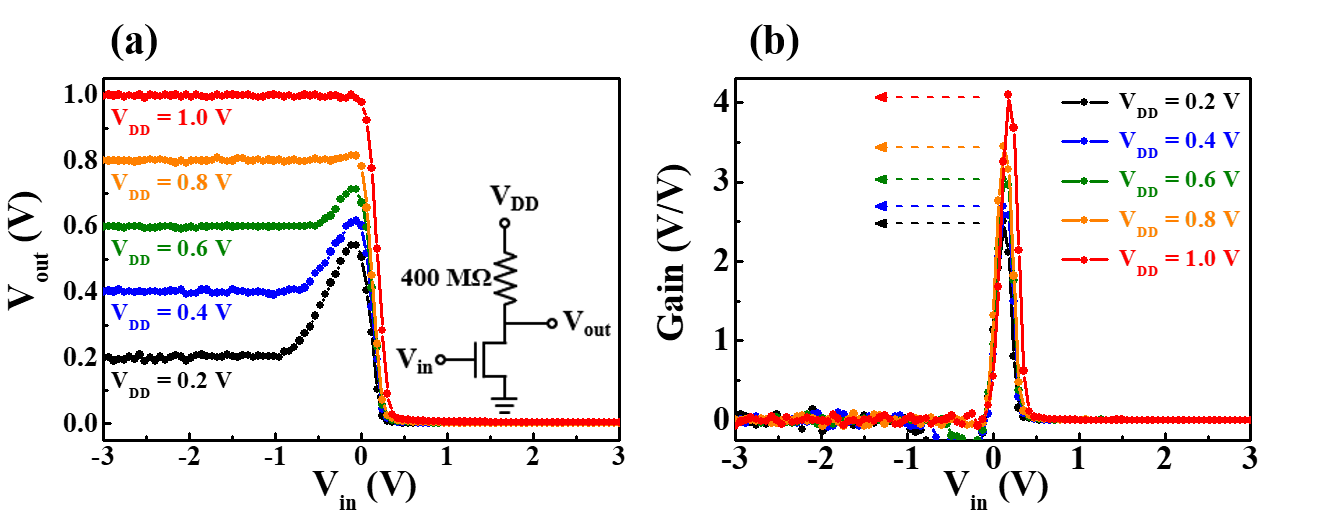


**Figure S5.** Resistor-loaded inverter characteristics of the chitosan electrolyte-Ta_2_O_5_ hybrid EDLT according to V_DD_ from 0.2 to 1.0 V (0.2 V steps) in which the 400 MΩ resistor was connected in series. (a) The VTC curves and the inset display the simple schematic of the equivalent circuit. (b) Calculated voltage gains (-dV_out_/dV_in_) from the VTC curves.

**Figure S6** shows the dynamic inverting response of the resistor (400 MΩ)-loaded chitosan electrolyte-Ta_2_O_5_ hybrid EDLT inverter. The dynamic inverting properties were evaluated by applying the low-voltage square-wave form input signal, which switches between –1 V and + 1 V at 1 Hz (**Figure S6a**) and 2 Hz (**Figure S6b**) to the gate-terminal (V_DD_ = 1 V). The chitosan electrolyte-Ta_2_O_5_ hybrid EDLT exhibits good dynamic inverting actions and small relaxation times along the order of milliseconds in the output signal.


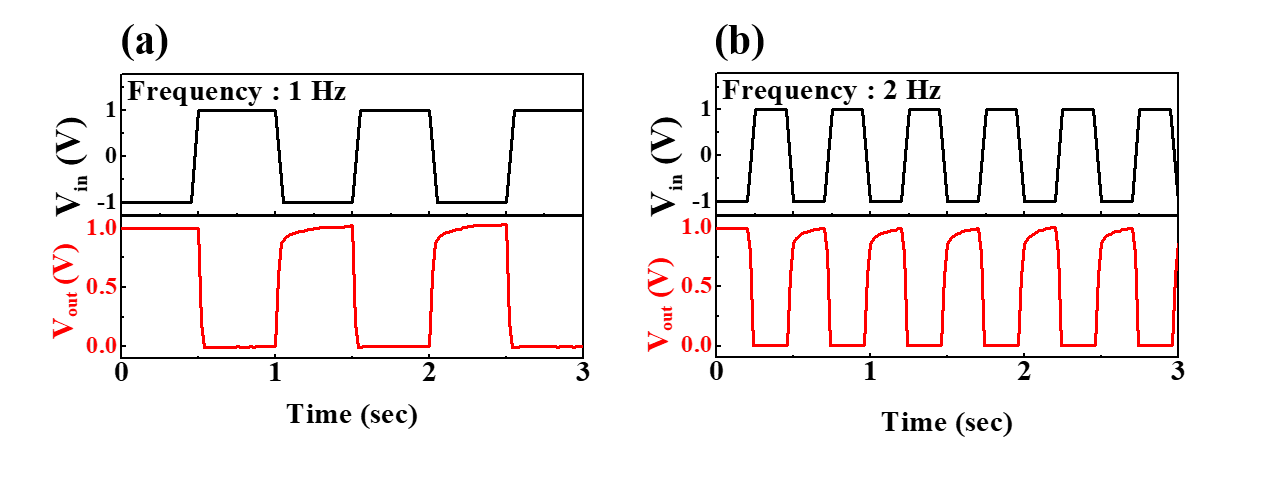


**Figure S6.** Dynamic inverting response properties for the resistor-loaded chitosan electrolyte-Ta_2_O_5_ hybrid EDLT inverter. The square-wave form input signals of the (a) 1 Hz and (b) 2 Hz frequency are applied on the gate-terminal.

**4. Excitatory post-synaptic current responses of the chitosan electrolyte-Ta_2_O_5_ hybrid EDLT**

**Figure S7** shows the excitatory post-synaptic current (EPSC) responses to the single pre-synaptic stimulus spike at different amplitudes (100 ms duration) of 0.5, 1, 2, 3, and 4 V. As the stimulus amplitude increased, the EPSC values also increased according to the amplitude and the inset shows the maximum EPSCs. When the spike stimulus ends, the EPSCs were rapidly decayed back by several milliseconds to the resting current value. This short EPSC duration time represents the short-term potentiation (STP).


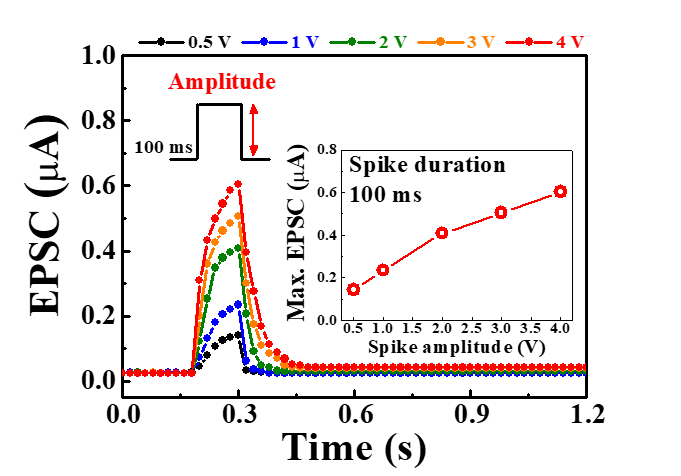


**Figure S7.** EPSC responses to the single pre-synaptic stimulus spikes at the different amplitudes of 0.5, 1, 2, 3, and 4 V (100 ms duration). The inset shows the maximum EPSC values according to the spike amplitude.

**Figure S8** shows the EPSC response according to the number of pre-synaptic stimulus spikes at different pulse amplitudes (1 V and 0.5 V) and different readout voltages (V_D_=1 V and 0.1 V). The pre-synaptic spike was applied at the bottom-gate electrode in the number of 1, 10, 30, 50, and 70 in 100 ms-width/10 ms-interval each pulse. **Figure S9** represents the maximum EPSC values after the pre-synaptic stimulus according to the pulse amplitudes, spike numbers, and readout voltages. The EPSC values were gradually increased according to the number of pre-synaptic spikes until the end of the stimulus. In addition, at low pulse amplitude and low readout voltage, the low magnitude of EPSC is steadily increased as following the stimulus number which indicates the mobile ions were successfully migrated in chitosan electrolyte at low-power consumption.


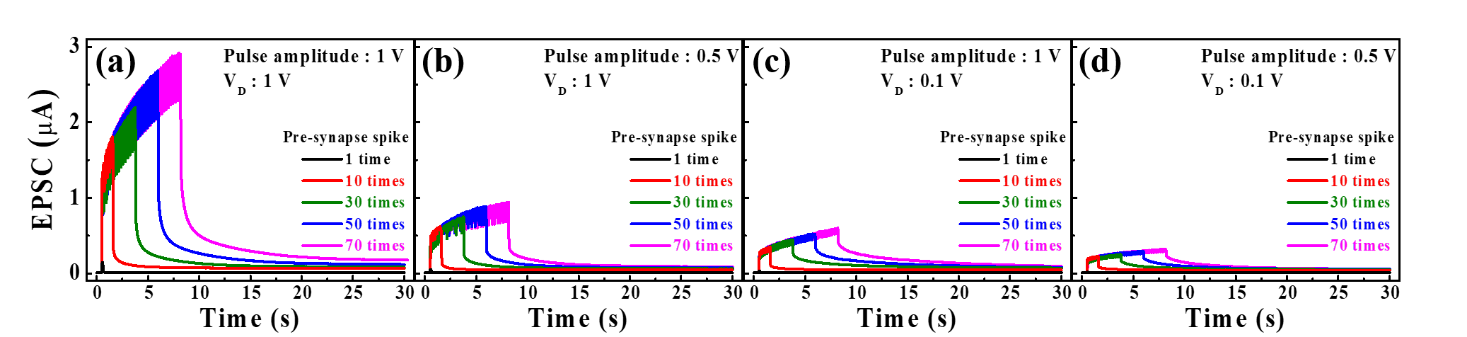


**Figure S8.** EPSC responses according to the number of pre-synaptic stimulus spikes (1, 10, 30, 50, and 70) at different pulse amplitudes (1 V and 0.5 V) and different readout voltages (V_D_=1 V and 0.1 V). Amplitude/V_D_: (a) 1 V/1 V, (b) 0.5 V/1 V, (c) 1 V/0.1 V, and (d) 0.5 V/ 0.1 V.


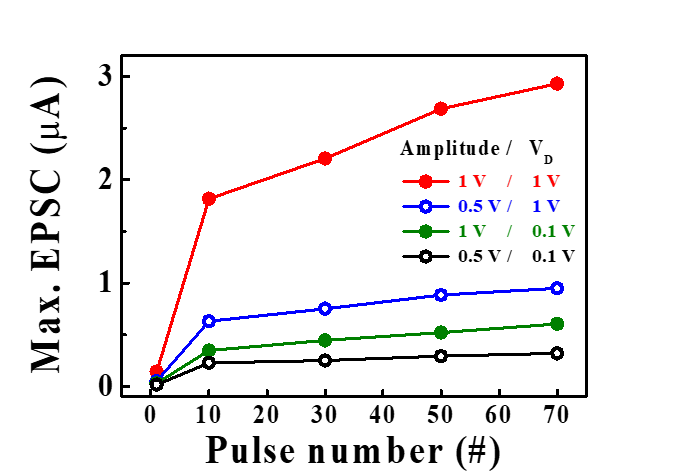


**Figure S9.** Maximum EPSC values after the pre-synaptic stimulus according to the pulse amplitudes, spike numbers, and readout voltages.

**5. Channel conductance modulation of the chitosan electrolyte-Ta_2_O_5_ hybrid EDLT**

**Figure S10** shows the continuous channel conductance modulation as a function of pre-synaptic pulse number in chitosan electrolyte-Ta_2_O_5_ hybrid EDLT. The continuous conductance increment (potentiation) and decrement (depression) were evaluated by applying repetitive positive pre-synaptic pulse (1.6 V for 10 ms) and negative pre-synaptic pulse (-1.8 V for 10 ms) at 0.1 V read voltage, respectively. In **Figure S10a**, chitosan electrolyte-Ta_2_O_5_ hybrid EDLT exhibits gradual conductance modulations at repetitive 3 × 10^2^ pre-synaptic pulses and magnified conductance modulations from 2 × 10^2^ to 3 × 10^2^ pulse stimulation is represented in **Figure S10b**. Through the repetitive positive and negative pre-synaptic pulses, the channel conductance is well-modulated in the dynamic range of ~ 5 nS.


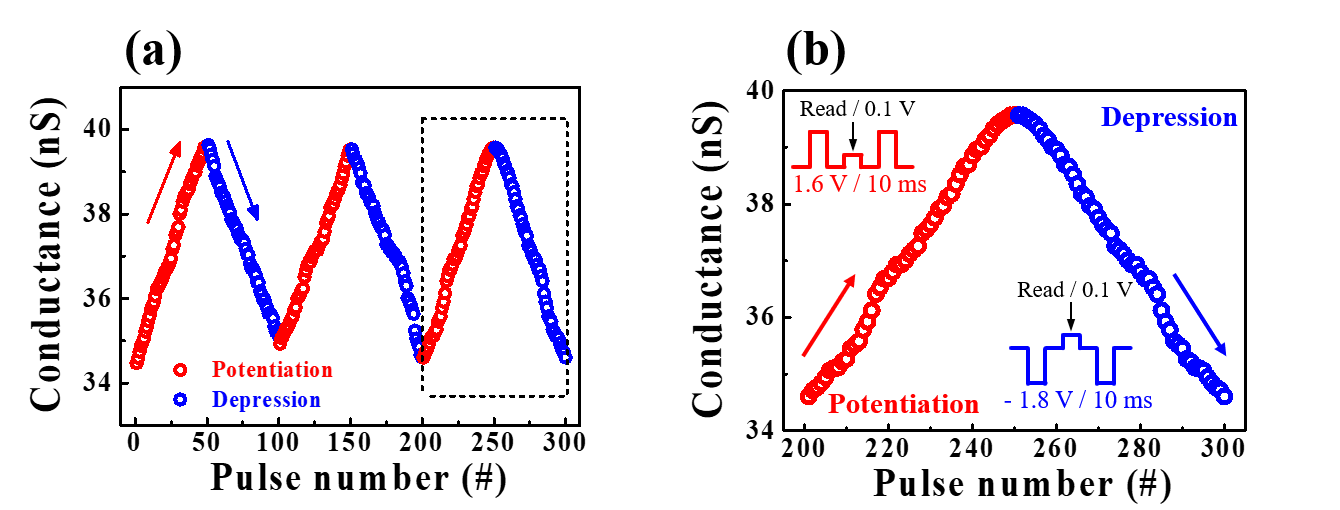


**Figure S10.** Gradual channel conductance modulation as a function of pre-synaptic pulse number in chitosan electrolyte-Ta_2_O_5_ hybrid EDLT. (a) Repetitive conductance potentiation and depression behaviours at 3 × 10^2^ pre-synaptic pulse stimulation. (b) Magnified conductance from 2 × 10^2^ to 3 × 10^2^ pulse stimulation and inset shows pulse scheme of potentiation (1.6 V for 10 ms)/depression (-1.8 V for 10 ms)/read (0.1 V).
